# Causal factors for osteoarthritis risk revealed by mendelian randomization analysis

**DOI:** 10.1007/s40520-024-02812-9

**Published:** 2024-08-22

**Authors:** Qingfeng Luo, Shiyong Zhang, Qiyuan Yang, Yuyi Deng, Hengjing Yi, Xingsheng Li

**Affiliations:** 1 State Key Laboratory of Ultrasound in Medicine and Engineering, Chongqing Medical University, Chongqing, 400016 China; 2 Chongqing Key Laboratory of Ultrasound Molecular Imaging, The Second Affiliated Hospital of Chongqing Medical University, Chongqing, 400010 China; 3 Department of Geriatrics, The Second Affiliated Hospital of Chongqing Medical University, Chongqing, 400010 China; 4 Department of Joint Surgery, The First Affiliated Hospital, Sun Yat-sen University, Guangzhou, 510080 Guangdong China

**Keywords:** Osteoarthritis, Mendelian randomization, Causality, Genetics

## Abstract

Osteoarthritis (OA), a prevalent chronic disease among the elderly, presents a complex pathogenesis and currently lacks effective treatment. Traditional observational studies are time-consuming, labor-intensive, susceptible to confounding factors, and cannot establish causal relationships. Mendelian randomization (MR) analysis, leveraging genetic variation to assess causal associations between exposures and outcomes, offers a cost-effective and efficient alternative. Over the past decade, large-scale genome-wide association studies have identified numerous genetic variants linked to OA risk factors, facilitating MR study design. In this review, we systematically identified 52 MR studies meeting specific criteria and evaluated their quality, exploring the impact of lifestyle, nutrition, comorbidities, circulating metabolites, plasma proteins, and other health factors on OA risk. We discuss the results and potential mechanisms of MR findings, addressing conflicting evidence based on existing literature and our prior research. With the ongoing expansion of genome-wide association data, we anticipate MR’s role in future OA studies to broaden, particularly in drug development research using targeted MR approaches. We thus aim for this paper to offer valuable insights for researchers and clinicians in related fields.

**Supplementary Information:**

The online version contains supplementary material available at 10.1007/s40520-024-02812-9.

## Introduction

Osteoarthritis (OA) is a common degenerative disease that primarily affects the elderly [1]. The pain and joint deformities associated with OA are significant contributors to disability and diminished quality of life in this age group [2]. The prevalence of osteoarthritis is increasing annually and is expected to increase by at least 50% overall by 2050 [3]. This has led to a significant rise in the costs associated with treatment and rehabilitation, posing a substantial public health burden and challenge [4–7]. Bone degeneration is a multifaceted process primarily influenced by genetic and environmental factors [8, 9]. Observational studies offer unparalleled advantages in conducting extensive epidemiological investigations and exploring causes. Nevertheless, in conventional observational analyses, it is challenging to fully address confounding factors [10]. Simultaneously, definitive proof of causal relationships between exposure factors and diseases remains elusive [11]. Findings on osteoarthritis in traditional observational studies are often disputed or conflicting, mainly due to ethical constraints and technical challenges. As a result, investigating the pathogenic factors and treatment strategies for osteoarthritis through Mendelian randomization (MR) analyses has emerged as a viable approach.

MR analysis is an observational study that uses genetic variation to make inferences about potential causal relationships between exposures (risk factors) and phenotypes [12, 13]. The genetic variants associated to exposure are randomly assigned at conception and are not influenced by factors such as acquired lifestyle [14]. Researchers consider MR to be akin to an observational randomized controlled trial, as it helps mitigate confounding factors and allows for the inference of causal associations found in observational studies [15]. Over the past 20 years, MR analyses have significantly advanced, expanding to investigate a wide range of topics. Initially focused on exploring risk factors for disease, these analyses now encompass studies on drug mechanisms of action and other relevant areas [14, 16, 17]. MR analysis mitigates ethical concerns by utilizing publicly available genetic variant data. These genetic variants are linked to exposure factors but remain unaffected by lifestyle or socioeconomic variables. The technical validity of Mendelian randomization studies has been extensively demonstrated across various degenerative conditions such as Alzheimer’s disease [18], Parkinson’s disease [19], and OA [20].

**Fig. 1:**
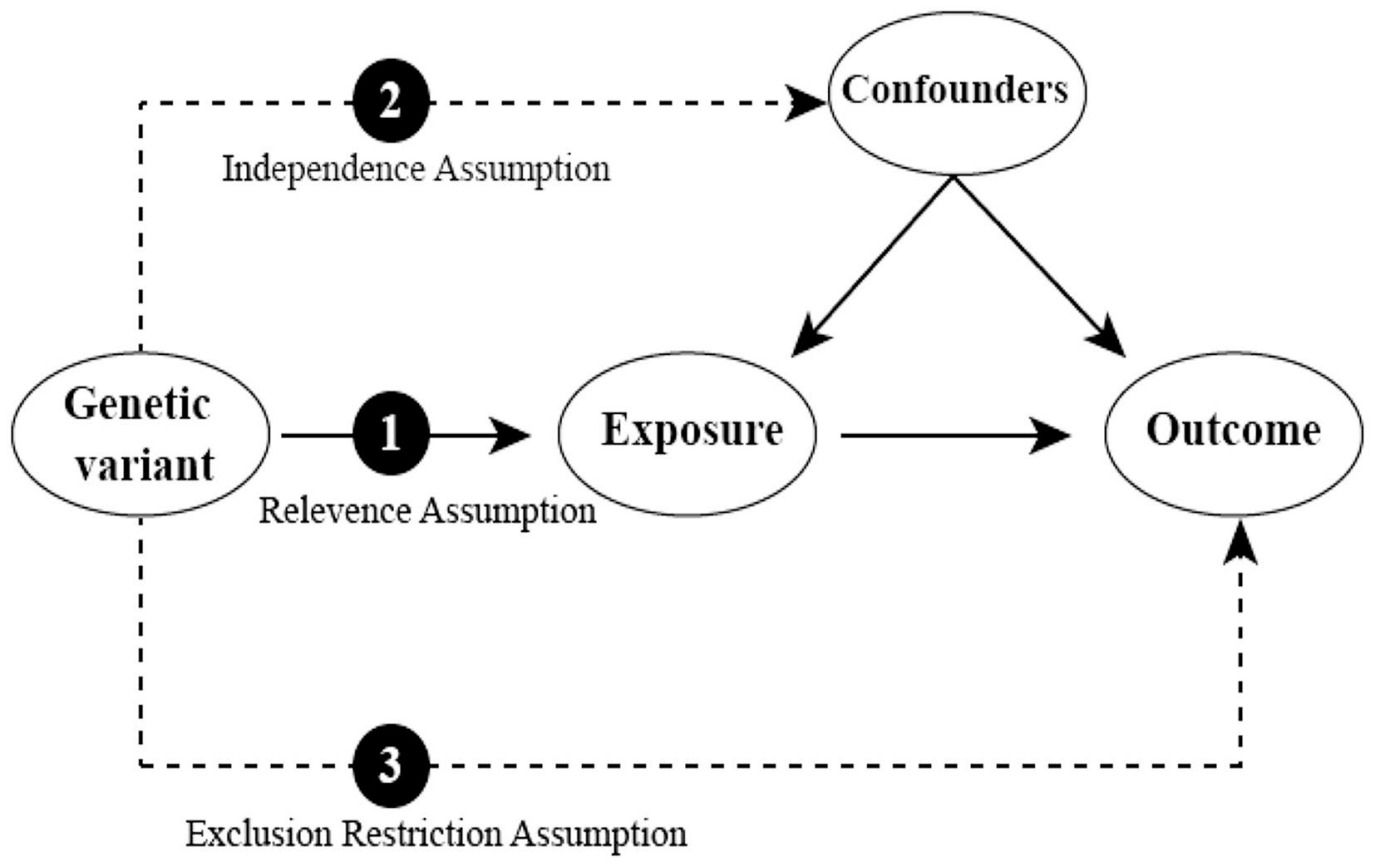
Basic principles and core assumptions of Mendelian randomization (MR). The relevance assumption asserts that genetic variation is strongly correlated with exposure. The independence assumption indicates that genetic variation is independent of potential confounding factors. Lastly, the exclusion restriction assumption proposes that genetic variation influences the outcome solely through its effect on exposure

The core assumptions of MR include the relevance assumption, meaning the genetic variants selected must have a strong association with the exposure; the independence assumption, meaning the genetic variants are not influenced by any measured or unmeasured confounders; and the exclusion restriction assumption, meaning the selected genetic variants can only affect the outcome through the exposure pathway (Fig. 1). In order for causal inferences between exposure and outcome to be valid, all three assumptions must be fully satisfied [21, 22]. The main methods used in MR analyses include single-sample MR analysis and two-sample MR analysis [23]. In single-sample MR, both exposure- and outcome-related genetic variants come from the same sample, allowing for the assessment of causality within a single population. On the other hand, two-sample MR analysis utilizes genetic variants for exposure and outcome from separate independent populations, which can increase the statistical efficacy of the method by utilizing existing pooled data from large-scale GWAS consortia [24, 25]. Various large biological databases currently offer genetic variation data, including the UK Biobank, which can support single-sample or two-sample MR analyses effectively. Moreover, a growing number of disease GWAS studies are enriching genetic data for multi-ethnic and multi-population MR analyses, significantly boosting both the quantity and quality of current MR studies [25, 26]. 

As in all specialties, exploring disease risk factors is necessary but often costly. MR analysis serves as a cost-effective and reliable research method that has significantly contributed to the body of evidence in OA research. Nevertheless, the number of risk factors for OA uncovered by MR is rapidly increasing, and certain causal associations appear to contradict available evidence, thereby complicating result interpretation. This review provides a comprehensive summary of MR studies related to OA published to date. A thorough search on PubMed (through December 2023), using specific keywords such as ‘Mendelian randomization,’ ‘osteoarthritis,’ ‘degenerative osteoarthropathy,’ and ‘OA,’ was conducted to identify relevant studies. The analysis encompasses a review of published reports on MR analyses in OA diseases, highlighting research progress and the challenges encountered.

## Results

### Description of included studies

**Fig. 2:**
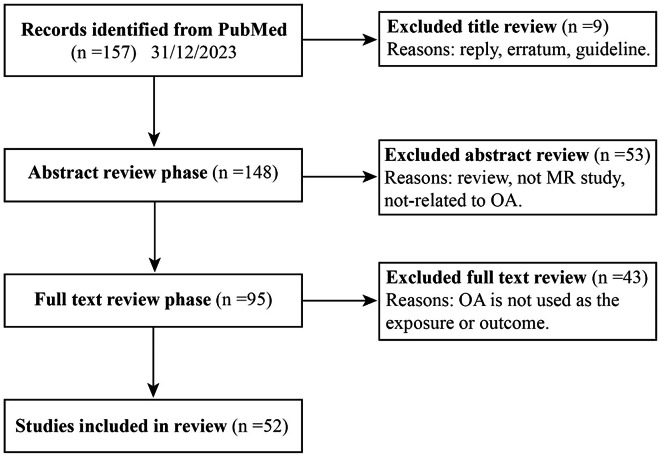
Literature screening process diagram

After applying several inclusion criteria, a total of 52 studies were included in this review (Fig. 2) [27–78]. Since there are no standardized quality assessment criteria, we followed the approach of several previous reviews and assessed the quality of these studies by examining their hypothesis validation [79, 80] (etable 1–3). Additionally, we summarized the main findings of these studies and indicated the source of SNPs for each study (etable 4). With a few exceptions, most studies used data from the UK Biobank, the largest human genetic database to date. Thus, MR studies of OA risk have been conducted mainly using genetic information from European populations. Factors revealed by MR to be associated with OA risk mainly include lifestyle, nutrition, comorbidities, circulating metabolites, plasma proteins, and other health factors. In several studies, OA has also been found to have a causal effect on other diseases when considered as an exposure factor. These associations were mainly found in reverse causality analyses of comorbidity MR studies, and we believe that these factors are also noteworthy for OA risk.

### Lifestyle and nutritional factors

The occurrence and development of osteoarthritis are influenced by both genetic and environmental factors [8, 9]. Numerous studies have highlighted the increasing incidence of osteoarthritis, which is closely linked to modern lifestyle changes [81](Table 1). Ni et al. reported a potential causal association between sleep and osteoarthritis, suggesting that insomnia or short sleep duration may increase the risk of osteoarthritis [27]. This finding is consistent with previous cross-sectional and case-control studies [82, 83], despite an incomplete understanding of the underlying biological mechanisms. Animal studies have reported that sleep deprivation can induce a cytokine storm-like syndrome in mammals, significantly weakening the body’s immunity [84]. Another perspective suggests that insufficient melatonin secretion due to sleep disorders may be a potential mechanism for these associations [85]. Melatonin is believed to affect OA by regulating proinflammatory factors, cartilage-degrading enzymes, and other inflammatory mediators [86]. Nevertheless, these insights currently lack validation in populations. Future exploration in large prospective cohorts will help identify these causal relationships and potential mechanisms.

The impact of exercise on osteoarthritis has been a topic of debate. Upon analyzing different exercise intensities, it was discovered that varying levels of exercise intensity had contrasting effects on osteoarthritis risk. Low-intensity exercise, as predicted genetically, was found to be associated with a higher risk of osteoarthritis [30], while moderate-intensity activities (such as housework and brisk walking) were associated with a reduced risk [28, 29]. In contrast to multiple intensities of exercise, a completely inactive lifestyle, such as sedentary behavior and prolonged television viewing, can significantly increase the risk of all osteoarthritis [31]. Although MR analysis revealed multiple causal associations between exercise and OA, observational studies remain skeptical of these associations [87], suggesting that the underlying mechanisms may be complex and unclear for a long time.

The debate surrounding the dietary impact of coffee and tea remains contentious. Various observational studies have highlighted a potential link between coffee and tea consumption and a lower risk of certain diseases, such as cancer, cardiovascular disease, and osteoarthritis [88–90]. However, a recent Mendelian randomization (MR) study suggested a possible association between genetically predicted coffee intake and a heightened risk of osteoarthritis [32], while tea consumption was found to significantly elevate the overall risk of osteoarthritis [33]. Such contradictory results may arise from study design, where observational studies are more likely to have biases. For example, our previous work found a dose-dependent, nonlinear association between coffee and tea consumption and bone health, and this association differed significantly by gender, coffee type, and other subgroups [91]. For these causal associations revealed by MR, some studies have reported that caffeine, the main active substance in coffee and tea, and its metabolites in vivo can directly influence the anabolic actions of chondrocytes [92, 93]. Moreover, it has been proposed that caffeine intake is associated with the inflammatory cytokines IL-1 and TNF-α [94]. Furthermore, consistent findings indicated that smoking and alcohol consumption were more prominently linked to an increased risk of osteoarthritis, with both behaviors significantly raising the risk across all types of osteoarthritis [34, 35].

**Table 1:** Evidence from MR studies on lifestyle and osteoarthritis risk

lifestyle	causal direction	Osteoarthritis	OR	years	Resources
Insomnia	→	Overall OA	1.22	2022	Ni [27].
Short sleep duration	→	Overall OA	1.04		Ni [27].
Housework	→	Overall OA	0.32	2023	Li [28].
	→	Knee OA	0.26		
Walking for pleasure	→	Overall OA	0.87		Li [28].
	→	Knee OA	0.14		
Walking pace	→	Overall OA	0.11	2023	Qiu [29].
	→	Knee OA	0.17		
	→	Hip OA	0.24		
leisure screen time	→	Overall OA	1.31	2023	Wang [30].
	→	Knee OA	1.44		
	→	Hip OA	1.13		
Sedentary behavior	→	Overall OA	1.80/1.98	2023	Cao [31]. /Li [28].
	→	Knee OA	1.85/1.64		
	→	Hip OA	1.65/1.32		
Coffee	→	Overall OA	1.01	2021	Zhang [32].
	→	Knee OA	1.02		
Tea	→	Overall OA	1.19	2022	Li [33].
Alcohol	→	Overall OA	1.33	2023	Wen [34].
Smoking	→	Overall OA	1.61	2022	Ni [35].
	→	Knee OA	1.54		
	→	Hip OA	1.62		

### Comorbidities

Comorbidities play a crucial role in predicting the onset and prognosis of osteoarthritis in research [95]. Observational studies have shown that the majority of osteoarthritis patients have at least one comorbid disease, often related to the cardiovascular and digestive systems [96, 97]. Recent MR studies have identified causal links between osteoarthritis and various comorbidities, although some studies present conflicting evidence. For example, several studies by Zhao et al. noted a causal association between genetically predicted risk of osteoarthritis and an increased risk of stroke [36–38], while Cai et al. suggested a causal association between ischemic stroke and osteoarthritis [39]. Additionally, Cai et al. identified a positive causal relationship between genetically predicted osteoarthritis and Parkinson’s disease [39]. Furthermore, regarding cardiovascular disease, the current MR evidence is contradictory. Wang et al. reported conflicting evidence, with hip osteoarthritis linked to an increased risk of heart failure, while coronary artery disease appeared to have a protective effect on knee osteoarthritis risk [37]. Xu et al. noted a causal association between myocardial infarction and reduced osteoarthritis risk [40]. Yin et al. highlighted a causal effect of osteoarthritis on atrial fibrillation risk but a protective effect against coronary atherosclerosis [41]. Other studies have shown that genetically determined osteoporosis may reduce osteoarthritis risk [42], while osteoarthritis could increase the risk of type 2 diabetes [44], gastroesophageal reflux disease [45], and bladder cancer [46], but no inverse association was found [43]. Moreover, allergic diseases like allergic rhinitis and asthma have been genetically linked to an increased risk of osteoarthritis [47](Table 2).

It is worth noting that caution should be exercised in interpreting causal associations between osteoarthritis and comorbidities. Some studies have suggested that the association between OA and the risk of cardiovascular disease can be explained by changes in the amount of exercise, but this view is not widely supported [98, 99]. In addition to potential confounders and study quality [45], co-inheritance, on the other hand, may explain some of the causal associations between diseases [100]. Although more mechanistic research evidence is needed to validate the association between osteoarthritis and comorbidities, this suggestive MR evidence could provide new directions for osteoarthritis research and consider more comorbidities in diagnosis and treatment.

**Table 2:** Evidence from MR studies on comorbidities and osteoarthritis risk

Comorbidities	Causal direction	Osteoarthritis (OA)	OR	Years	Resources
Stroke	←	Overall OA	1.12	2022	Zhao [36].
	←	Hip OA	1.14	2022	Wang [37].
Lacunar stroke	←	Hip OA	1.20	2022	Shen [38].
Ischemic stroke	→	Overall OA	1.03	2021	Cai [39].
Parkinson’s disease	←	Overall OA	1.19		Cai [39].
Heart failure	←	Hip OA	1.07	2022	Wang [37].
Coronary heart disease	→	Knee OA	0.90		Wang [37].
Myocardial infarction	→	Overall OA	0.95	2022	Xu [40].
Atrial fibrillation	←	Overall OA	1.11	2023	Yin [41].
Coronary Atherosclerosis	← →	Overall OA	0.88 0.95		Yin [41].
Osteoporosis	→	Overall OA	0.75	2022	Liu [42].
Type 2 diabetes	→	Overall OA	—	2020	Cui [43].
Type 2 diabetes	←	Knee OA	1.18	2023	Xing [44].
Gastroesophageal Reflux disease	←	Overall OA	1.26	2023	Xu [45].
Bladder cancer	←	Overall OA	1.07	2023	Zhang [46].
Allergic disease	→	Knee OA	1.07	2023	Baker [47].
Allergic rhinitis	→	Knee OA	1.07		Baker [47].
Asthma	→	Overall OA	1.02		Baker [47].
	→	Knee OA	1.04		

### Circulating metabolites and proteins

Osteoarthritis is a multifaceted process involving metabolism, nutrition, and inflammation [101–103].While traditional observational studies have examined various peripheral biomarkers and established their potential link to the risk of osteoarthritis, most of these markers are assessed at specific time points in both cross-sectional and cohort studies. This makes it challenging to determine the association between longitudinal trends over time and osteoarthritis. Consequently, there has been a growing interest in MR studies in recent years, which aim to investigate the causal relationship between genetically predicted levels of peripheral markers and the risk of osteoarthritis.

The role of metabolism in the progression of bone and joint health has been extensively studied, with abnormal metabolites potentially acting as predictive markers for osteoarthritis and even exacerbating the disease [101, 104]. Recent advancements in large-scale next-generation sequencer technology and GWAS analysis have facilitated the study of metabolism-related Mendelian randomization analysis. Through 11 MR studies, causal relationships between thousands of metabolites and the risk of osteoarthritis have been investigated [48–58](Table 3). Eleven metabolites were found to have a causal association with an increased risk of osteoarthritis, including isovaleryl carnitine, taurocholate, kynurenine, acetaminophen 4-sulfate, homocysteine, serum iron, transferrin saturation, insulin-like growth factor-1, serum copper, and serum zinc. On the other hand, 10 metabolites were associated with a reduced risk of osteoarthritis, such as 1-linolenoylglycerophosphorylcholine, arginine, alanine (Ala), omega-3 fatty acids, omega-6 fatty acids, LDL cholesterol, transferrin, folate, vitamin B12, and serum calcium. Potential mechanistic explanations include direct effects of metabolites on chondrocytes, or altering the inflammatory state, which in turn affects the metabolic activity of cartilage. For example, nitric oxide, a metabolite produced in vivo from arginine, has strong antioxidant and anti-inflammatory effects, and oxidative stress has been widely implicated in the progression of OA [105, 106]. In contrast, kynurenine, a major degradation product of tryptophan, inhibits chondrocyte cell proliferation in a dose-dependent manner [107]. Furthermore, activation of the kynurenine-aromatic hydrocarbon receptor axis impairs chondrogenesis and chondroprotection in mesenchymal stromal cells [108]. Similarly, one of the components of Omega-6 fatty acids, arachidonic acid (AA), has been identified as a precursor to a variety of potent pro-inflammatory mediators. Consequently, Omega-6 fatty acid levels are significantly correlated with chronic inflammation in the body [109, 110].

Although a number of other metabolites have been found to be causally related to OA, the underlying mechanisms remain unclear. For instance, two separate MR studies have shown a link between increased LDL cholesterol levels and a reduced risk of osteoarthritis, contradicting previous observational studies [52, 53]. It has been suggested that LDL can reduce APOA1 levels and serum amyloid A protein in human primary chondrocytes and fibroblast-like synoviocytes, thereby alleviating joint inflammation [111]. However, we remain cautious about this hypothesis. While the specific mechanisms of action of these metabolites on osteoarthritis were not determined by the researchers, their findings offer valuable insights for future studies.

**Table 3:** Evidence from MR studies on metabolism-related molecules and osteoarthritis risk

Metabolism-related molecules	Causal direction	Osteoarthritis (OA)	OR	Years	Resources
1-linoleoylglycerophosphocholine	→	Hand/Finger OA	0.36/0.21	2023	Gu [48].
Isovalerylcarnitine	→	Hand OA	1.51		Gu [48].
Taurocholate	→	Thumb OA	1.35		Gu [48].
Kynurenine	→	Hip/Knee OA	1.44		Gu [48].
Arginine	→	Hip OA	0.56		Gu [48].
4-acetaminophen sulfate	→	Hip OA	1.02		Gu [48].
Alanine(Ala)	→	Hip/Knee OA	0.82	2023	Cui [49].
Homocysteine	→	Overall OA	1.10	2023	Hong [50].
	→	Knee OA	1.08		Hong [50].
	→	Hip OA	1.08		Hong [50].
Omega−3 fatty acids	→	Knee OA	0.94	2023	Li [51].
Omega−6 fatty acids	→	Knee OA	0.93		Li [51].
	→	Hip OA	0.89		Li [51].
Low-density lipoprotein cholesterol(LDL)	→	Overall OA	0.87	2019	Hindy [52].
Low-density lipoprotein cholesterol(LDL)	→	Hip/Knee OA	0.90	2022	Meng [53].
Apolipoprotein B(APOB)	→	Hip/Knee OA	0.93		Meng [53].
	→	Hip OA	0.89		Meng [53].
	→	Knee OA	0.93		Meng [53].
Serum iron	→	Hip OA	1.18	2023	Ruan [54].
Transferrin saturation	→	Hip OA	1.15		Ruan [54].
Transferrin saturation	→	Knee OA	1.07	2022	Xu [55].
	→	Hip OA	1.16		
	→	Hip/Knee OA	1.09		
Transferrin	→	Hip OA	0.92	2022	Xu [55].
	→	Hip/Knee OA	0.95		
Folate	→	Overall OA	0.87	2023	Hong [50].
Vitamin B12	→	Knee OA	0.95		Hong [50].
Insulin-like growth factor−1 (IGF−1)	→	Hip OA	1.57	2021	Hartley [56].
	→	Knee OA	1.30		
Serum copper	→	Overall OA	1.07	2021	Zhou [57].
Serum zinc	→	Overall OA	1.18		Zhou [57].
	→	Others OA	1.21		
Serum calcium	→	Overall OA	0.71	2021	Qu [58].
	→	Hip OA	0.53		
	→	Knee OA	0.64		

Research has demonstrated a strong association between diseases such as thyroid disorders and gonadal dysfunction with the development of osteoarthritis [112, 113]. Twelve studies have identified potential causal links between 14 protein and hormone molecular biomarkers and the risk of osteoarthritis [48, 58–67, 78](Table 4). Eight markers were found to be associated with an increased risk of osteoarthritis, including serum testosterone, serum dihydrotestosterone, sex hormone-binding globulin, glycosylated hemoglobin (HbA1c), insulin-like growth factor-binding protein 4, lipocalin, leptin, and resistin. On the other hand, six markers were genetically predictive of causality and linked to a reduced risk of osteoarthritis, such as X-11,423-O-sulfo-L-tyrosine, ADpSGEGDFXAEGGGVR, parathyroid hormone, and retinol. Some studies provide further support for existing observational evidence. For instance, elevated levels of parathyroid hormone have been shown to be causally linked to a decreased risk of osteoarthritis [60]. Previous research has demonstrated that parathyroid hormone-related proteins inhibit the hypertrophic differentiation of chondrocytes, which is beneficial for repairing cartilage damage and osteoarthritis [114]. In addition, animal studies have confirmed that PTH slows the progression of cartilage degeneration in OA mice by reducing the number of mast cells in the subchondral bone and maintaining its microstructure [115]. Mendelian randomization analyses also support this genetic correlation, offering potential targets for future interventions.

The association between glycosylated hemoglobin (HbA1c) and insulin-like growth factor-binding protein 4 with an increased risk of osteoarthritis validates the link between diabetes and osteoarthritis risk [65, 66]. Potential mechanisms include local toxicity from high glucose exposure, increased expression of cytokines and protein hydrolases, and accumulation of advanced glycation end products (AGEs). Previous studies have reported that high glucose levels promote cartilage degeneration by increasing the expression of cyclooxygenase−2 (COX−2) and MMP−13, while decreasing the synthesis of type II collagen and peroxisome proliferator-activated receptor γ (PPARγ) [116]. Rasheed et al. further showed that AGEs induce IL−6 and IL−8 expression in human OA chondrocytes through the receptor for AGEs (RAGE) activation pathway, leading to oxidative stress [117]. We believe that the effects of hyperglycemia on OA are multifaceted due to its ability to induce a wide range of metabolic disorders.

MR reveals a causal relationship between adipokines, such as adipose transport proteins, leptin, and resistin, and the risk of osteoarthritis; however, the underlying mechanisms are more complex. Zhao et al. reported that high-dose leptin induces cell cycle arrest and senescence in chondrogenic progenitor cells through activation of the p53/p21 pathway and inhibition of the Sirt1 pathway, thereby promoting OA [118]. Moreover, Feng et al. reviewed the mechanisms of lipocalin’s effects on OA, suggesting that lipocalin may regulate chondrocyte autophagy and promote cellular pyroptosis in OA through multiple pathways [119]. On the other hand, it has been noted that while adipokines may trigger chondrocyte apoptosis, they primarily act as inflammatory mediators [120].

**Table 4:** Evidence from MR studies on protein, hormone molecules and osteoarthritis risk

Protein & hormone molecules	Causal direction	Osteoarthritis (OA)	OR	Years	Resources
X−11,423–O-sulfo-L-tyrosine	→	Hand OA	0.44	2023	Gu [48].
ADpSGEGDFXAEGGGVR	→	Hip OA	0.65		Gu [48].
Parathyroid hormone	→	Knee OA	0.53	2021	Qu [59].
Parathyroid hormone	→	Overall OA	0.73	2021	Huang [60].
Serum retinol	→	Hip OA	0.44	2021	Qu [58].
Testosterone (T)	→	Hip OA	1.56	2021	Yan [61].
Dihydrotestosterone (DHT)	→	Hip OA	1.39		Yan [61].
Sex-hormone-binding-globulin (SHBG)	→	Overall OA	1.09	2020	Qu [62].
Alanine transaminase (ALT)	→	Hip OA	2.48	2023	Huang [63].
	→	Knee OA	3.07		Huang [63].
Retinol	→	Hip OA	0.45		Huang [64].
Glycated hemoglobin (HbA1c)	→	Knee OA	1.56	2023	Chen [65].
Insulin-like-growth factor-binding-protein4(IGFBP4)	→	Overall OA	1.48	2023	Han [66].
	→	Knee OA	2.15		
Adiponectin	→	Knee OA	1.28	2021	Fan [67].
Leptin	→	Knee OA	3.44		Fan [67].
Resistin	→	Knee OA	1.18		Fan [67].

Numerous studies have confirmed the presence of chronic low-grade inflammation in the progression of osteoarthritis [121]. Traditional observational studies have shown that elevated levels of systemic and local inflammation play a role in the progression of osteoarthritis. Additionally, abnormal peripheral inflammatory factors have been found to be somewhat predictive of the trajectory of osteoarthritis [122]. Two MR studies have demonstrated a causal link between genetically predicted levels of inflammatory factors and the risk of osteoarthritis [66, 78]. The expression of immune-related CD25-associated traits has been linked to a lower risk of osteoarthritis [69]. Additionally, lesser-known factors such as macrophage inflammatory protein−1β and tumor necrosis factor beta have also shown potential in reducing the risk of osteoarthritis [68](Table 5). By extrapolating the association between these genetically predicted inflammatory factors and osteoarthritis risk, we can gain a deeper understanding of the biological mechanisms underlying inflammation in osteoarthritis.

**Table 5:** Evidence from MR studies on immune, inflammatory markers and osteoarthritis risk

Immune & inflammatory markers	Causal direction	Osteoarthritis (OA)	OR	Years	Resources
Macrophage-inflammatory protein−1beta (MIP−1β)	→	Overall OA	0.99	2023	Su [68].
Tumour necrosis factor beta (TNF-β)	→	Overall OA	0.99		Su [68].
C-C motif chemokine ligand 5(CCL5)	→	Overall OA	1.01		Su [68].
Macrophage-colony-stimulating-factor (MCSF)	→	Knee OA	1.16	2023	Huang [78].
Vascular-endothelial-growth-factor (VEGF)	→	Knee OA	1.09		Huang [78].
CD25-related traits (7个)	→	Hip OA	0.8–0.9	2023	Luo [69].
IgG4 Kappa(ig γ-4)	→	Overall OA	1.38	2023	Han [66].
	→	Knee OA	1.89		

### Other health factors

Due to the intricate pathogenesis of osteoarthritis, numerous MR studies have investigated potential connections between osteoarthritis and various health indicators such as childhood obesity, sarcopenia, body mass index, age at menstruation and childbearing, blood pressure, bone density, and gut flora [70–77](Table 6). The association between childhood obesity and adult health has been supported by numerous studies, including those focusing on osteoarthritis. A 25-year cohort study revealed a significant link between childhood overweight and knee pain, stiffness, and dysfunction in adulthood [123]. MR studies have confirmed a genetically predictable causal relationship between muscle loss or underweight and significant metabolic disturbances [70], which can lead to deterioration in general health. While the association with osteoarthritis has not received much attention [124], MR studies have found a significant causal link between genetically determined muscle loss and an increased risk of all osteoarthritis [71]. An abnormal rise in basal metabolism is often indicative of endocrine disruption or nutritional imbalance, a systemic alteration that has been associated with the risk for a variety of diseases [125]. Several previous MR studies have found a causal association between genetically predicted elevations in basal metabolic rate and osteoporosis and several cancers [126, 127]. Similarly, in osteoarthritis, MR studies have validated genetically predicted causal connections [73]. Further research is needed to understand the mechanisms and clinical implications of protective factors like age at menarche/first childbearing and gut flora in osteoarthritis [71, 77].

Interestingly, conflicting evidence was found in multiple MR studies regarding the relationship between bone mineral density (BMD) and osteoarthritis. Funck et al. reported a positive causal relationship between increased femoral neck BMD and increased risk of osteoarthritis, with each standard deviation increase in BMD increasing the risk of overall osteoarthritis, hip osteoarthritis, and knee osteoarthritis by 14%, 22%, and 18%, respectively [74]. Jiang et al. supported this finding by showing that genetically higher whole-body BMD increased the risk of hip osteoarthritis [76]. In addition, Liu et al. also noted that osteoporosis (decreased BMD) had a genetically predicted protective effect against osteoarthritis [42]. Some studies have proposed the hypothesis that increased bone density is associated with subchondral bone sclerosis and increased bone resorption [128, 129]. We are cautious about this hypothesis. In contrast, Qu et al. found that genetically reduced BMD in specific areas was associated with an increased risk of osteoarthritis [75]. The potential mechanistic explanation for this association is that bone loss in the subchondral bone in patients with OP leads to articular surface collapse and uneven articular cartilage stress, resulting in secondary osteophyte proliferation and cartilage damage [130]. Overall, the association between BMD and OA remains controversial. We believe that these conflicting results stem primarily from differences in the instrumental variables selected for MR studies.

**Table 6:** Evidence from MR studies on other health factors and osteoarthritis risk

Health factors	Causal direction	Osteoarthritis (OA)	OR	Years	Resources
Childhood obesity	→	Overall OA	1.01	2022	Cao [70].
	→	Hip OA	1.13		
	→	Knee OA	1.11		
Appendicular-lean-mass(ALM)	→	Hip OA	1.15	2023	Yang [71].
	→	Knee OA	1.10		
Age at menarche(AAM)	→	Knee OA	0.86	2022	Wang [72].
Age at first birth (AFB)	→	Overall OA	0.80		Wang [72].
	→	Hip/Knee OA	0.79		
Basal metabolic rate (BMR)	→	Overall OA	1.01	2023	Zhou [73].
	→	Hip OA	1.47		
	→	Knee OA	1.87		
Low systolic BP	→	Overall OA	1.55	2019	Funck [74].
Body mass index(BMI)	→	Overall OA	1.57		Funck [74].
	→	Hip OA	1.52		
	→	Knee OA	1.76		
High femoral neck BMD	→	Overall OA	1.14		Funck [74].
	→	Hip OA	1.22		
	→	Knee OA	1.18		
Low femoral neck BMD	→	Hip OA	1.12	2023	Qu [75].
	→	Knee OA	1.11		
Low lumber spine BMD	→	Overall OA	1.05		Qu [75].
	→	Hip OA	1.16		
	→	Knee OA	1.11		
total body bone mineral density (TB-BMD)	→	Hip OA	1.20	2022	Jiang [76].
Methanobacteriaceae family	→	Knee OA	0.93	2021	Yu [77].
	→	Overall OA	0.95		
Desulfovibrionales order	→	Knee OA	0.88		Yu [77].
Ruminiclostridium5 genus	→	Knee OA	0.85		Yu [77].

### Future research directions

Current MR analyses provide extensive evidence of OA risk, at least in a statistically significant manner. Regarding the need for additional MR studies, we believe that future research should focus on MR analyses that help develop treatment options for OA, such as drug-targeted MR.

Drug-targeted MR studies, an emerging research method, aim to evaluate whether changing exposure through therapeutic manipulation of a target can lead to expected outcomes [131–133]. In the field of osteoarthritis drug development, MR analysis is being used to investigate therapeutic mechanisms and identify specific targets. For instance, metformin, a well-known drug used for diabetes treatment, has shown protective effects against osteoarthritis [134, 135]. Research indicates that metformin may protect cartilage by targeting cells or activating specific receptors [136, 137]. Drug-targeted MR analyses have further confirmed that metformin targets AMP-activated protein kinase (AMPK) and growth differentiation factor 15 (GDF-15) have genetically predicted protective effects against osteoarthritis [138]. This strengthens the evidence for developing targeted therapies for osteoarthritis.

Combining MR with other methodologies, such as multi-omics analysis or MR-based multi-omics data analysis, can significantly improve result confidence. Additionally, given that most MR analyses are currently conducted using genetic data from European populations, there is a need to perform them in other ethnicities and subpopulations in the future to enhance result generalizability. Finally, causal associations revealed by MR should not solely rely on statistical significance but should be complemented with experimental studies to validate the functional roles of identified genetic variants and pathways in OA pathogenesis.

### Limitations

However, we should be aware that MR analysis has its limitations in advancing disease research, particularly in reporting or interpreting MR results [139]. Common pitfalls include chain imbalance [13], horizontal pleiotropy [140], vertical pleiotropy [141], weak instrumental variables [142], and population stratification [143]. It is essential to carefully consider these shortcomings to ensure more accurate results when conducting or interpreting MR analyses. In addition, we should also note that observational studies have limitations that may deviate from real-world scenarios, even if the analysis process and interpretation of the results are fully compliant with norms. For example, in a drug-targeted MR analysis, the authors found a causal association between genetic variants in PCSK9 inhibitors and statins and the risk of cognitive impairment [144]. Ference et al. argued that the results of this study were unreliable in the real world, as PCSK9 monoclonal antibodies do not cross the blood-brain barrier, and also contradicted previous MR reports [145]. It is crucial to recognize that while observational studies can guide research direction and provide a broader perspective, statistically significant associations may not always align with actual outcomes.

## Conclusion

In conclusion, MR analysis offers valuable insights into OA risk research. MR’s research model, based on genetic information, minimizes confounding factors and ethical concerns. In the exploration of OA risk, MR can efficiently and rapidly reveal potential associations and provide causal evidence. The application of drug-targeted MR further enhances the utility of MR analysis in OA therapeutic research, offering precise guidance for drug development. Looking ahead, there is a growing trend towards integrating MR with other methods, which can enhance result confidence. Additionally, adhering to rigorous research methodology and reporting standards is crucial for improving the quality and reliability of MR studies in the field of OA.

## Electronic supplementary material

Below is the link to the electronic supplementary material.

Supplementary Material 1

## Data Availability

No datasets were generated or analysed during the current study.
