# Supplementary material for: Causal factors for osteoarthritis risk revealed by mendelian randomization analysis
Source: Aging Clin Exp Res. 2024 Aug 22;36(1):176. doi: 10.1007/s40520-024-02812-9 (PMC11341639; doi:10.1007/s40520-024-02812-9)
Supplement: Supplementary file 1 — Supplementary Material 1 [file 40520_2024_2812_MOESM1_ESM.docx]

**Supplementary material**

**Causal factors for osteoarthritis risk revealed by Mendelian randomization analysis**

**etable 1**. Criteria for evaluating the quality of included studies.

| **Classification** | **Criteria** |
| --- | --- |
| Good | • **Relevance Assumption**: Strong association between instrument variables and exposure (F-statistic > 10, genome-wide significant p-value < 5E-8), with detailed correlation analysis.  • **Independence Assumption**: Thorough handling of potential confounders, including PhenoScanner, article research, or multiple variable MR.  • **Horizontal Pleiotropy Assumption**: Application of multiple methods (e.g., MR-Egger, MR-PRESSO) to detect and correct pleiotropy, with detailed reporting of heterogeneity tests. |
| Moderate | • **Relevance Assumption**: Moderate association between instrument variables and exposure (F-statistic close to 10, genome-wide significant p-value < 1E-5), with basic correlation analysis.  • **Independence Assumption**: Basic handling of potential confounders, including PhenoScanner, article research, or multiple variable MR.  • **Horizontal Pleiotropy Assumption**: Some pleiotropy detection methods applied, but may be incomplete or not detailed. |
| Poor | • **Relevance Assumption**: Weak association between instrument variables and exposure (F-statistic < 10, genome-wide significant p-value not available), with insufficient correlation analysis.  • **Independence Assumption**: Inadequate handling of potential confounders, lacking multiple sample and sensitivity analyses.  • **Horizontal Pleiotropy Assumption**: No pleiotropy detection or correction methods applied, and heterogeneity tests not reported. |

**etable 2**. Criteria for evaluating the generalizability of findings.

| **Classification** | **Criteria** |
| --- | --- |
| Good | • **Population Diversity**: Study sample includes a wide diversity of age, sex, ethnicity, and geographic regions.  • **Analysis**: Thorough analysis of gene-environment interactions across different demographic groups.  • **Replication**: Results have been validated in independent samples and different populations.  • **Reporting**: Detailed reporting of demographic characteristics and subgroup analyses, with consistent findings across groups. |
| Poor | • **Population Diversity**: Study sample lacks diversity in age, sex, ethnicity, or geographic regions.  • **Analysis**: Limited or no analysis of gene-environment interactions across different demographic groups.  • **Replication**: Results have not been validated in independent samples or different populations.  • **Reporting**: Insufficient reporting of demographic characteristics and lack of subgroup analyses, with inconsistent or unreported findings across groups. |

**etable 3**. Assessment of the quality and generalizability of the included studies.

| **Authors** | **Exposure** | **Relevance** | | **Independence** | **Horizontal Pleiotropy** | **Quality** | **Generalizability** |
| --- | --- | --- | --- | --- | --- | --- | --- |
|  |  | **F-statistic** | **p-value** |  |  |  |  |
| Ni.^[1]^ | Insomnia, sleep duration | > 10 | < 5E-8 | multiple variable MR | MR-PRESSO,  MR-Egger | Good | Good |
| Li.^[2]^ | Housework | > 10 | < 5E-8 | NA | MR-PRESSO,  MR-Egger | Moderate | Good |
| Qiu.^[3]^ | Walking pace | > 10 | < 5E-8 | multiple variable MR | MR-PRESSO,  MR-Egger | Good | Good |
| Wang.^[4]^ | Leisure screen time | NA | < 5E-8 | PhenoScanncer | MR-PRESSO,  MR-Egger | Good | Good |
| Cao.^[5]^ | Leisure sedentary behavior | NA | < 5E-8 | PhenoScanncer | MR-PRESSO,  MR-Egger | Good | Good |
| Zhang.^[6]^ | Coffee | NA | < 5E-8 | PhenoScanncer | MR-PRESSO,  MR-Egger | Good | Good |
| Li.^[7]^ | Tea | > 10 | < 5E-8 | NA | NA | Moderate | Good |
| Wen.^[8]^ | Alcohol | > 10 | NA | PhenoScanncer | MR-PRESSO,  MR-Egger | Good | Good |
| Ni.^[9]^ | Smoking | > 10 | < 5E-8 | multiple variable MR | MR-PRESSO,  MR-Egger | Good | Good |
| Zhao.^[10]^ | Stroke | > 10 | < 5E-8 | NA | MR-PRESSO,  MR-Egger | Moderate | Good |
| Wang.^[11]^ | CAD | > 10 | < 5E-8 | NA | MR-PRESSO,  MR-Egger | Moderate | Good |
| Shen.^[12]^ | Lacunar stroke | > 10 | < 5E-8 | NA | MR-PRESSO,  MR-Egger | Moderate | Good |
| Cai.^[13]^ | Parkinson's disease | > 10 | < 5E-8 | NA | MR-Egger | Moderate | Good |
| Xu.^[14]^ | Myocardial infarction | > 10 | < 5E-8 | PhenoScanncer | MR-PRESSO,  MR-Egger | Good | Good |
| Yin.^[15]^ | Atrial fibrillation, CAD | > 10 | < 5E-8 | NA | MR-Egger | Moderate | Good |
| Liu.^[16]^ | Osteoporosis | > 10 | < 5E-8 | NA | MR-PRESSO,  MR-Egger | Moderate | Good |
| Cui.^[17]^ | Type 2 diabetes | > 10 | < 5E-8 | NA | MR-Egger | Moderate | Good |
| [Xing](https://pubmed.ncbi.nlm.nih.gov/?size=50&term=Xing+X&cauthor_id=37525375).^[18]^ | Type 2 diabetes | > 10 | < 5E-8 | PhenoScanncer | MR-PRESSO,  MR-Egger | Good | Good |
| Xu.^[19]^ | gastroesophageal reflux disease | > 10 | < 5E-8 | PhenoScanncer | MR-PRESSO,  MR-Egger | Good | Good |
| Zhang.^[20]^ | Bladder cancer | > 10 | < 5E-8 | PhenoScanncer | MR-PRESSO,  MR-Egger | Good | Good |
| Baker.^[21]^ | Asthma | NA | < 5E-8 | NA | MR-Egger | Moderate | Good |
| Gu.^[22]^ | Circulation metabolites | > 10 | < 5E-8 | NA | MR-PRESSO,  MR-Egger | Moderate | Good |
| Cui.^[23]^ | Serum AA | NA | <5E-04 | NA | MR-PRESSO,  MR-Egger | Moderate | Good |
| Hong.^[24]^ | Homocysteine, folate, Vit-B12 | NA | < 5E-8 | PhenoScanncer | MR-PRESSO,  MR-Egger | Good | Good |
| Li.^[25]^ | Polyunsaturated fatty acid | > 10 | < 5E-8 | NA | MR-Egger | Moderate | Good |
| Hindy.^[26]^ | LDL, BMI | NA | < 5E-8 | NA | MR-Egger | Moderate | Good |
| Meng.^[27]^ | LDL, APOB | NA | NA | NA | MR-PRESSO,  MR-Egger | Moderate | Good |
| Ruan.^[28]^ | iron status | NA | < 5E-8 | PhenoScanncer | MR-PRESSO,  MR-Egger | Good | Good |
| Xu.^[29]^ | iron status | NA | < 5E-8 | PhenoScanncer | MR-Egger | Good | Good |
| Hartley^[30]^ | IGF-1 | NA | NA | multiple variable MR | MR-Egger | Moderate | Good |
| Zhou^[31]^ | Copper, zinc | > 10 | < 5E-8 | NA | MR-Egger | Moderate | Good |
| Qu.^[32]^ | Calcium | NA | < 5E-8 | NA | MR-PRESSO,  MR-Egger | Moderate | Good |
| Qu.^[33]^ | PTH | NA | < 5E-8 | NA | MR-PRESSO,  MR-Egger | Moderate | Good |
| Huang.^[34]^ | PTH | > 10 | < 5E-8 | NA | MR-PRESSO,  MR-Egger | Moderate | Good |
| Yan.^[35]^ | Testosterone, DHT | NA | < 5E-8 | PhenoScanncer | MR-PRESSO,  MR-Egger | Good | Good |
| Qu.^[36]^ | SHBG | NA | < 5E-8 | PhenoScanncer | MR-PRESSO,  MR-Egger | Good | Good |
| Huang.^[37]^ | ALT | > 10 | < 5E-8 | PhenoScanncer | MR-Egger | Good | Good |
| Huang.^[38]^ | Circulating antioxidants | > 10 | < 5E-8 | NA | MR-PRESSO,  MR-Egger | Moderate | Good |
| Chen.^[39]^ | HbA1c | > 10 | < 5E-8 | NA | MR-PRESSO,  MR-Egger | Moderate | Good |
| Han.^[40]^ | Plasma proteins | > 10 | < 5E-8 | NA | MR-Egger | Moderate | Good |
| Fan.^[41]^ | Adipokine | > 10 | < 5E-8 | PhenoScanncer | MR-Egger | Good | Good |
| Su.^[42]^ | Cytokines | > 10 | < 5E-8 | NA | MR-PRESSO,  MR-Egger | Moderate | Good |
| Huang.^[43]^ | inflammatory cytokines | > 10 | < 5E-8 | NA | MR-PRESSO,  MR-Egger | Moderate | Good |
| Luo.^[44]^ | Immune factors | > 10 | < 5E-8 | PhenoScanncer | MR-Egger | Good | Good |
| Cao.^[45]^ | Childhood obesity | > 10 | < 5E-8 | NA | MR-PRESSO,  MR-Egger | Moderate | Good |
| Yang.^[46]^ | Sarcopenia | > 10 | < 5E-8 | PhenoScanncer | MR-PRESSO,  MR-Egger | Good | Good |
| Wang.^[47]^ | Reproductive factors | > 10 | < 5E-8 | NA | MR-Egger | Moderate | Good |
| Zhou.^[48]^ | Basal metabolic rate | > 10 | < 5E-8 | PhenoScanncer | MR-PRESSO,  MR-Egger | Good | Good |
| Funck.^[49]^ | BMI, BMD, blood pressure | NA | < 1E-5 | NA | MR-Egger | Moderate | Good |
| Qu.^[50]^ | BMD | NA | < 5E-8 | NA | MR-Egger | Moderate | Good |
| Jiang.^[51]^ | BMD | NA | < 5E-8 | PhenoScanncer | MR-Egger | Good | Good |
| Yu.^[52]^ | Gut microbiota | > 10 | < 1E-5 | NA | MR-Egger | Moderate | Good |

**etable 4**. Primary findings and data sources of outcomes for included studies.

| **Authors** | **Exposure** | **Findings** | **Data sources** |
| --- | --- | --- | --- |
| Ni.^[1]^ | Insomnia, Short sleep duration | Findings suggested an adverse effect of insomnia or short sleep duration on OA risk. | UKB,  23andMe |
| Li.^[2]^ | Housework | Engaging in light DIY activities and walking for pleasure were beneficial for preventing OA, while prolonged television watching increased the risk of OA. | UKB, Genetics of Osteoarthritis (GO) Consortium |
| Qiu.^[3]^ | Walking pace | WP could reduce the risk of OA and its subtypes. | UKB |
| Wang.^[4]^ | Leisure screen time | Reducing LST had a protective effect on OA. | Genetics of Osteoarthritis (GO) Consortium |
| Cao.^[5]^ | leisure sedentary behavior | Prolonged TV watching was causally associated with an increased risk of OA. | FinnGen Biobank, UKB, BioBank Japan |
| Zhang.^[6]^ | Coffee | Coffee consumption exerted a causal effect on OA risk. | UKB |
| Li.^[7]^ | Tea | High daily tea intake could increase the risk of OA. | MRC-IEU, UKB |
| Wen.^[8]^ | Alcohol | Alcohol intake frequency was a risk factor for OA. | GLGC, MRC-IEU, UKB, arcOGEN |
| Ni.^[9]^ | Smoking | Findings support an independent deleterious causal effect of smoking on OA risk. | GSCAN, GIANT, 23andMe, UKB |
| Zhao.^[10]^ | Stroke | Hip OA is a potential risk factor for stroke. | UKB, arcOGEN |
| Wang.^[11]^ | CVD | The risk of OA was reduced in patients with heart disease, but the risk of heart failure and stroke was increased in patients with OA. | IEU |
| Shen.^[12]^ | Lacunar stroke | OA had a causal effect on the risk of LS. | arcOGEN |
| Cai.^[13]^ | Parkinson's disease | OA had a positive causal effect on PD. | UKB |
| Xu.^[14]^ | Myocardial infarction | MI could be potentially protective against OA risk. | Genetics of Osteoarthritis (GO) Consortium |
| Yin.^[15]^ | Atrial fibrillation, CA. | OA was a risk factor for AF, and there was a mutual protective factor between OA and CA. | ArcOGEN, UKB |
| Liu.^[16]^ | Osteoporosis | OP could reduce the incidence of OA. | UKB |
| Cui.^[17]^ | T2D | Findings did not support causal effects of T2D risk on OA risk. | UKB, arcOGEN, IEU |
| [Xing](https://pubmed.ncbi.nlm.nih.gov/?size=50&term=Xing+X&cauthor_id=37525375).^[18]^ | T2D | OA could be a potential risk factor for T2D. | UKB, arcOGEN, IEU |
| Xu.^[19]^ | gastroesophageal reflux disease (GORD) | OA has an adverse effect on GORD, which is partly attributed to opioid use in patients with OA. | UKB, arcOGEN |
| Zhang.^[20]^ | Bladder cancer | A positive causal relationship has been found between OA and bladder cancer risk. | FinnGen, UKB |
| Baker.^[21]^ | Asthma | Findings support a causal relationship between asthma and OA. | UKB |
| Gu.^[22]^ | Circulation metabolites | Nine strong pathogenic associations between seven metabolites and OA were found. | TwinsUK, KORA, meta-analysis of 9 different populations |
| Cui.^[23]^ | Serum AA | Genetically predicted alanine (Ala), tyrosine (Tyr) and isoleucine (Ile) levels were significantly associated with OA risk. | meta-analysis of 9 different populations |
| Hong.^[24]^ | Homocysteine, folate, vitamin B12 | Homocysteine had adverse effects on OA; Folate and vitamin B12 had protective effects on OA. | meta-analysis of 9 different populations |
| Li.^[25]^ | Polyunsaturated fatty acid (PUFA) | High omega-6 fatty acids could reduce the risk of OA. | UKB |
| Hindy.^[26]^ | LDL,BMI | Elevation in LDL level was associated with a lower risk of OA; elevation in BMI was associated with an increased risk of OA. | UKB |
| Meng.^[27]^ | LDL, APOB | APOB and LDL had protective effects on OA. | UKB |
| Ruan.^[28]^ | iron status | High iron status might be a causal factor of OA. | meta-analysis of 9 different populations |
| Xu.^[29]^ | iron status | Transferrin saturation had a positive causal relationship with OA, whereas transferrin had a negative causal relationship with OA. | UKB, arcOGEN |
| Hartley^[30]^ | IGF-1 | Increased serum IGF-1 was causally related to higher risk of OA. | UKB, arcOGEN |
| Zhou^[31]^ | Copper and zinc | High zinc and copper status were positively associated with OA risk. | UKB |
| Qu.^[32]^ | Calcium | A negative causal relationship has been found between serum calcium levels and the risk of OA. | UKB |
| Qu.^[33]^ | PTH | Serum PTH concentration was negatively associated with the development of OA. | UKB, arcOGEN |
| Huang.^[34]^ | Parathyroid hormone (PTH) | A potential causal relationship existed between reduced serum PTH and increased risk of OA. | UKB, arcOGEN |
| Yan.^[35]^ | Testosterone (T), DHT | Serum T and DHT levels may play causal roles in the development of OA. | UKB, arcOGEN |
| Qu.^[36]^ | SHBG | There were positive causal effects of circulating SHBG on the development of OA. | UKB |
| Huang.^[37]^ | ALT | ALP was linked to a higher risk of OA. | Million Veteran Program, UKB |
| Huang.^[38]^ | Circulating antioxidants | Absolute circulating levels of retinol was associated with a reduced risk of OA. | UKB, arcOGEN |
| Chen.^[39]^ | HbA1c | Increased HbA1c levels had a causal effect on the risk of OA. | IEU |
| Han.^[40]^ | Plasma proteins | IgG4 Kappa (ig gamma-4) and IGFBP4 (insulin-like growth factor-binding protein 4) had causal effects on OA. | meta-analysis of 9 different populations |
| Fan.^[41]^ | Adipokine | Lipocalin, leptin, and resistin levels were causally associated with increased OA risk. | UKB |
| Su.^[42]^ | Cytokines | Macrophage inflammatory protein-1β (MIP-1β) and tumor necrosis factor beta (TNF-β) levels were causally associated with OA risk; C-C motif chemokine ligand 5 (CCL5) had a suggestive association with OA risk. | UKB |
| Huang.^[43]^ | inflammatory cytokines | Inflammatory cytokines, namely MCSF and VEGF, were causally associated with OA. | UKB |
| Luo.^[44]^ | Immune factors | CD25, especially in CD4CD25 T cells, exerted a protective influence against the development of OA. | UKB, arcOGEN |
| Cao.^[45]^ | Childhood obesity (CO) | There appears to be a causal relationship between childhood obesity and OA. | UKB |
| Yang.^[46]^ | Sarcopenia (SP) | SP may have a causal effect on OA. | meta-analysis of 9 different populations |
| Wang.^[47]^ | Reproductive factors | Age at first menarche (AAM) and age at first birth (AFB) have a causal effect on the OA risk. | UKB |
| Zhou.^[48]^ | BMR | There was a potential causal relationship between BMR and OA risk. | UKB, arcOGEN |
| Funck.^[49]^ | BMI, BMD, BP | BMI ,BMD and BP exhibited a significant causal effect on OA risk. | UKB |
| Qu.^[50]^ | BMD | OP may be causally linked to an increased risk of OA. | UKB |
| Jiang.^[51]^ | BMD | Systematically higher TB-BMD was associated with the risk of OA. | UKB, arcOGEN |
| Yu.^[52]^ | Gut microbiota | Several microbial taxa were causally associated with diverse joint OA. | UKB, arcOGEN |

**Reference**

[1] Ni J; Zhou W; Cen H, et al. Evidence for causal effects of sleep disturbances on risk for osteoarthritis: a univariable and multivariable Mendelian randomization study. *Osteoarthritis Cartilage*.**2022,** *30* (3), 443-450.

[2] Li X; Wang S; Liu W, et al. Causal effect of physical activity and sedentary behaviors on the risk of osteoarthritis: a univariate and multivariate Mendelian randomization study. *Sci Rep*.**2023,** *13* (1), 19410.

[3] Qiu P; Wu J; Kui L, et al. Causal effects of walking pace on osteoarthritis: a two-sample mendelian randomization study. *Front Genet*.**2023,** *14*, 1266158.

[4] Wang B; Liu Y; Zhang YC, et al. Assessment of causal effects of physical activity on the risk of osteoarthritis: a two-sample Mendelian randomization study. *BMC Med Genomics*.**2023,** *16* (1), 237.

[5] Cao Z; Li Q; Li Y, et al. Causal association of leisure sedentary behavior with arthritis: A Mendelian randomization analysis. *Semin Arthritis Rheum*.**2023,** *59*, 152171.

[6] Zhang Y; Fan J; Chen L, et al. Causal Association of Coffee Consumption and Total, Knee, Hip and Self-Reported Osteoarthritis: A Mendelian Randomization Study. *Front Endocrinol (Lausanne)*.**2021,** *12*, 768529.

[7] Li G; Zhang Z; Liu Y. Genetically predicted tea intake increases the risk of osteoarthritis: A Mendelian randomization study. *Front Genet*.**2022,** *13*, 1004392.

[8] Wen MT; Liang XZ; Luo D, et al. Plasma lipids, alcohol intake frequency and risk of Osteoarthritis: a Mendelian randomization study. *BMC Public Health*.**2023,** *23* (1), 1327.

[9] Ni J; Wang P; Yin KJ, et al. Does smoking protect against developing osteoarthritis? Evidence from a genetically informed perspective. *Semin Arthritis Rheum*.**2022,** *55*, 152013.

[10] Zhao H; Zhu J; Ju L, et al. Osteoarthritis & stroke: a bidirectional mendelian randomization study. *Osteoarthritis Cartilage*.**2022,** *30* (10), 1390-1397.

[11] Wang Z; Kang C; Xu P, et al. Osteoarthritis and cardiovascular disease: A Mendelian randomization study. *Front Cardiovasc Med*.**2022,** *9*, 1025063.

[12] Shen Y; Li F; Cao L, et al. Hip Osteoarthritis and the Risk of Lacunar Stroke: A Two-Sample Mendelian Randomization Study. *Genes (Basel)*.**2022,** *13* (9).

[13] Cai Y; Zhang G; Liang J, et al. Causal Relationships Between Osteoarthritis and Senile Central Nerve System Dysfunction: A Bidirectional Two-Sample Mendelian Randomization Study. *Front Aging Neurosci*.**2021,** *13*, 793023.

[14] Xu H; Ling Y; Jiang H, et al. Osteoarthritis, coronary artery disease, and myocardial infarction: A mendelian randomization study. *Front Cardiovasc Med*.**2022,** *9*, 892742.

[15] Yin M; Xu W; Pang J, et al. Causal relationship between osteoarthritis with atrial fibrillation and coronary atherosclerosis: a bidirectional Mendelian randomization study of European ancestry. *Front Cardiovasc Med*.**2023,** *10*, 1213672.

[16] Lin L; Luo P; Yang M, et al. Causal relationship between osteoporosis and osteoarthritis: A two-sample Mendelian randomized study. *Front Endocrinol (Lausanne)*.**2022,** *13*, 1011246.

[17] Cui Z; Feng H; He B, et al. Type 2 Diabetes and Glycemic Traits Are Not Causal Factors of Osteoarthritis: A Two-Sample Mendelian Randomization Analysis. *Front Genet*.**2020,** *11*, 597876.

[18] Xing X; Wang Y; Pan F, et al. Osteoarthritis and risk of type 2 diabetes: A two-sample Mendelian randomization analysis. *J Diabetes*.**2023,** *15* (11), 987-993.

[19] Xu H; Wei J; Chen D, et al. Assessing causality between osteoarthritis and gastrointestinal disorders: a Mendelian randomization study. *Sci Rep*.**2023,** *13* (1), 19603.

[20] Zhang X; Wen Z; Xing Z, et al. The causal relationship between osteoarthritis and bladder cancer: A Mendelian randomization study. *Cancer Med*.**2023,** *13* (1).

[21] Baker MC; Robinson WH; Ostrom Q. Genetic association between atopic disease and osteoarthritis. *Osteoarthritis Cartilage*.**2023,** *32* (2), 220-225.

[22] Gu Y; Jin Q; Hu J, et al. Causality of genetically determined metabolites and metabolic pathways on osteoarthritis: a two-sample mendelian randomization study. *J Transl Med*.**2023,** *21* (1), 357.

[23] Cui ZY; Feng H; He BC, et al. Causal associations between serum amino acid levels and osteoarthritis: a Mendelian randomization study. *Osteoarthritis Cartilage*.**2023,** *31* (8), 1121-1131.

[24] Hong H; Chen L; Zhong Y, et al. Associations of Homocysteine, Folate, and Vitamin B12 with Osteoarthritis: A Mendelian Randomization Study. *Nutrients*.**2023,** *15* (7).

[25] Li X; Lu Z; Qi Y, et al. The Role of Polyunsaturated Fatty Acids in Osteoarthritis: Insights from a Mendelian Randomization Study. *Nutrients*.**2023,** *15* (22).

[26] Hindy G; Åkesson KE; Melander O, et al. Cardiometabolic Polygenic Risk Scores and Osteoarthritis Outcomes: A Mendelian Randomization Study Using Data From the Malmö Diet and Cancer Study and the UK Biobank. *Arthritis Rheumatol*.**2019,** *71* (6), 925-934.

[27] Meng H; Jiang L; Song Z, et al. Causal Associations of Circulating Lipids with Osteoarthritis: A Bidirectional Mendelian Randomization Study. *Nutrients*.**2022,** *14* (7).

[28] Ruan G; Ying Y; Lu S, et al. The effect of systemic iron status on osteoarthritis: A mendelian randomization study. *Front Genet*.**2023,** *14*, 1122955.

[29] Xu J; Zhang S; Tian Y, et al. Genetic Causal Association between Iron Status and Osteoarthritis: A Two-Sample Mendelian Randomization. *Nutrients*.**2022,** *14* (18).

[30] Hartley A; Sanderson E; Paternoster L, et al. Mendelian randomization provides evidence for a causal effect of higher serum IGF-1 concentration on risk of hip and knee osteoarthritis. *Rheumatology (Oxford)*.**2021,** *60* (4), 1676-1686.

[31] Zhou J; Liu C; Sun Y, et al. Genetically predicted circulating levels of copper and zinc are associated with osteoarthritis but not with rheumatoid arthritis. *Osteoarthritis Cartilage*.**2021,** *29* (7), 1029-1035.

[32] Qu Z; Yang F; Hong J, et al. Causal relationship of serum nutritional factors with osteoarthritis: a Mendelian randomization study. *Rheumatology (Oxford)*.**2021,** *60* (5), 2383-2390.

[33] Qu Z; Yang F; Yan Y, et al. A Mendelian randomization study on the role of serum parathyroid hormone and 25-hydroxyvitamin D in osteoarthritis. *Osteoarthritis Cartilage*.**2021,** *29* (9), 1282-1290.

[34] Huang G; Zhong Y; Li W, et al. Causal Relationship Between Parathyroid Hormone and the Risk of Osteoarthritis: A Mendelian Randomization Study. *Front Genet*.**2021,** *12*, 686939.

[35] Yan YS; Qu Z; Yu DQ, et al. Sex Steroids and Osteoarthritis: A Mendelian Randomization Study. *Front Endocrinol (Lausanne)*.**2021,** *12*, 683226.

[36] Qu Z; Huang J; Yang F, et al. Sex hormone-binding globulin and arthritis: a Mendelian randomization study. *Arthritis Res Ther*.**2020,** *22* (1), 118.

[37] Huang G; Li W; Zhong Y, et al. Mendelian randomization to evaluate the causal relationship between liver enzymes and the risk of six specific bone and joint-related diseases. *Front Immunol*.**2023,** *14*, 1195553.

[38] Huang L; Xie Y; Jin T, et al. Diet-derived circulating antioxidants and risk of knee osteoarthritis, hip osteoarthritis and rheumatoid arthritis: a two-sample Mendelian randomization study. *Front Med (Lausanne)*.**2023,** *10*, 1147365.

[39] Chen L; Jia C; Yang H. Causal Effect of Higher Glycated Hemoglobin (HbA1c) Levels on Knee Osteoarthritis Risk: A Mendelian Randomization Study. *Rheumatol Ther*.**2023,** *10* (1), 239-247.

[40] Han BX; Yan SS; Yu H, et al. Causal Effects of Plasma Proteome on Osteoporosis and Osteoarthritis. *Calcif Tissue Int*.**2023,** *112* (3), 350-358.

[41] Fan J; Zhu J; Sun L, et al. Causal association of adipokines with osteoarthritis: a Mendelian randomization study. *Rheumatology (Oxford)*.**2021,** *60* (6), 2808-2815.

[42] Su D; Ai Y; Zhu G, et al. Genetically predicted circulating levels of cytokines and the risk of osteoarthritis: A mendelian randomization study. *Front Genet*.**2023,** *14*, 1131198.

[43] Huang G; Li W; Kan H, et al. Genetic influences of the effect of circulating inflammatory cytokines on osteoarthritis in humans. *Osteoarthritis Cartilage*.**2023,** *31* (8), 1047-1055.

[44] Luo H; Zhu Y; Guo B, et al. Causal relationships between CD25 on immune cells and hip osteoarthritis. *Front Immunol*.**2023,** *14*, 1247710.

[45] Cao Z; Wu Y; Li Q, et al. A causal relationship between childhood obesity and risk of osteoarthritis: results from a two-sample Mendelian randomization analysis. *Ann Med*.**2022,** *54* (1), 1636-1645.

[46] Yang J; Liu P; Wang S, et al. Causal relationship between sarcopenia and osteoarthritis: a bi-directional two-sample mendelian randomized study. *Eur J Med Res*.**2023,** *28* (1), 327.

[47] Wang B; Wu J; Li H, et al. Using genetic instruments to estimate the causal effect of hormonal reproductive factors on osteoarthritis. *Front Public Health*.**2022,** *10*, 941067.

[48] Zhou J; Wei P; Yi F, et al. The association between basal metabolic rate and osteoarthritis: a Mendelian randomization study. *BMC Med Genomics*.**2023,** *16* (1), 258.

[49] Funck-Brentano T; Nethander M; Movérare-Skrtic S, et al. Causal Factors for Knee, Hip, and Hand Osteoarthritis: A Mendelian Randomization Study in the UK Biobank. *Arthritis Rheumatol*.**2019,** *71* (10), 1634-1641.

[50] Qu Y; Chen S; Han M, et al. Osteoporosis and osteoarthritis: a bi-directional Mendelian randomization study. *Arthritis Res Ther*.**2023,** *25* (1), 242.

[51] Jiang L; Jiang Y; Wang A, et al. The causal association between bone mineral density and risk of osteoarthritis: A Mendelian randomization study. *Front Endocrinol (Lausanne)*.**2022,** *13*, 1021083.

[52] Yu XH; Yang YQ; Cao RR, et al. The causal role of gut microbiota in development of osteoarthritis. *Osteoarthritis Cartilage*.**2021,** *29* (12), 1741-1750.
